# Genome-wide and molecular evolution analysis of the Poplar KT/HAK/KUP potassium transporter gene family

**DOI:** 10.1002/ece3.299

**Published:** 2012-07-19

**Authors:** Caiyun He, Kai Cui, Aiguo Duan, Yanfei Zeng, Jianguo Zhang

**Affiliations:** State Key Laboratory of Tree Genetics and Breeding, Key Laboratory of Silviculture of the State Forestry Administration, Research Institute of Forestry, Chinese Academy of ForestryBeijing, 100091, People's Republic of China

**Keywords:** Asymmetric evolutionary rates, KT/HAK/KUP family, segmental duplication, tandem duplication.

## Abstract

As the largest K^+^ transport gene family, KT/HAK/KUP family plays an important role in plant growth, development, and stress adaptation. However, there is limited information about this family in woody plant species. In this study, with genome-wide in-depth investigation, 31 Poplar KT/HAK/KUP transporter genes including six pairs of tandem duplicated and eight pairs of segmental duplicated paralogs have been identified, suggesting segmental and tandem duplication events contributed to the expansion of this family in Poplar. The combination of phylogenetic, exon structure and splice site, and paragon analysis revealed 11 pairs of Poplar KT/HAK/KUP duplicates. For these 11 pairs, all pairs are subject to purify selection, and asymmetric evolutionary rates have been found to occur in three pairs. This study might provide more insights into the underlying evolution mechanisms of trees acclimating to their natural habitat.

## Introduction

Gene family is a set of homologous genes that are likely to share similar functions. Changes in family size due to lineage-specific gene duplication and gene loss might provide insights into the evolutionary forces that have shaped eukaryotic genomes(Demuth et al. #b^501^). Thus, inferring an evolutionary scenario for a gene family is essential for understanding the phenotypic diversification of organisms (Doyon and Liu#b^502^).

The K^+^ transport systems are multifunctional proteins encoded by a large gene family found in nearly all plant tissues. The critical K^+^ requirement for plants and the complex composition of plant environments make the number of K^+^ transport systems so large to enable the uptake of K^+^ from the soil and its redistribution throughout diverse plant tissues (Gierth et al. 2005; Ashley et al. 2006; Amrutha et al. 2007). In plants, potassium transporters have been classified into four major families: the KT/HAK/KUP family, Trk/HKT family, KEA (K^+^ efflux anti-porter) family, and CHX (cation/hydrogen exchanger) family (Gupta et al. 2008). Among them, KT/HAK/KUP family is the largest and it plays an important role in plant growth and development (Ahn et al. 2004). The first plant *KT/HAK/KUP* gene was identified in Arabidopsis by sequence similarity with bacteria KUP and fungi HAKs (Quintero and Blatt 1997; Santa-Maria et al. 1997). To date, *KT/HAK/KUP* genes have been cloned from many plant species such as pepper (Martinez-Cordero et al. 2004), tomato (Wang et al. 2002; Nieves-Cordones et al. 2007), lotus (Desbrosses et al. 2004), grapevine (Davies et al. 2006), and seagrass (Garciadeblas et al. 2002). Twenty-seven and 13 *KT/HAK/KUP* family genes have been identified based on genome-wide sequence analysis in rice (*Oryza sativa*; Yang et al. 2009) and *Arabidopsis thaliana* (Maser et al. 2001), respectively. Lineage-specific expansion of this HAK family in rice after the split of monocots and dicots has been found. However, limited information is available on *KT/HAK/KUP* genes in perennial species. Such long-lived species are often exposed to repeated episodes of abiotic and biotic stresses during their long life span (Brosche et al. 2005). These features might have favored the expansion of *KT/HAK/KUP* gene family in perennial species.

The complex history of genome duplications and chromosomal rearrangements in Populus, and the recently available genome sequences provides an opportunity to study the patterns of gene family expansion in the course of genome evolution (Tuskan et al. 2006). In this study, we first identified the *KT/HAK/KUP* transporters gene family members in Poplar and then integrated analysis of phylogeny, exon structure, protein domain, and paralogon were applied to elucidate the evolutionary relationships of Poplar *KT/HAK/KUP* transporters genes. In addition, modes of selection were measured for the *KT/HAK/KUP* duplicate pairs, and a relative rate test was also conducted to examine whether one of the duplicates has evolved at an accelerated rate following the duplication. With such in-depth investigation, it might provide more insights into the underlying evolution mechanisms of trees acclimating to their natural habitat.

## Methods

### Genomic data mining, *KT/HAK/KUP* gene identification and nomenclature

To identify *KT/HAK/KUP* genes in *Populus trichocarpa*, 13 full-length *KT/HAK/KUP* protein sequences of *Arabidopsis thaliana* (Maser et al. 2001; Very and Sentenac 2003) (Table S1) were used as the query sequences in TBLASTN searches (E < 1e-10) against *P. trichocarpa* genome database (JGI v2.0). Each matching sequences were used reiteratively to search the respective sequence databases, until no new sequences were found. Then, the tool of Pfam (Sonnhammer et al. 1997) was used to predict the potassium (K^+^) transporter domain (PF02705) of all the candidate proteins. The deduced nucleotide and protein sequences of *KT/HAK/KUP* genes in *P. trichocarpa* were downloaded from the 31st release of the Gramene database (http://www.gramene.org/). The EST searches for *KT/HAK/KUP* genes in *P. trichocarpa* were performed using BLASTN tool against the EST database in PopulusDB. The nomenclature for Populus *KT/HAK/KUP*s followed the study of Yang et al. (2009).

### Phylogenetic analysis

To gain more insight into evolution of the *KT/HAK/KUP* gene family in *P. trichocarpa*, we downloaded the members of this family in green alga (*Chlamydomonas reinhardtii*) and rice (*Oryza sativa* L.). Multiple amino acid sequence alignments of *KT/HAK/KUP*s were generated using muscle3.6 (Edgar 2004) with the default setting. The bootstrap consensus phylogenetic tree inferred from 1000 replicates was constructed using the neighbor-joining method (Saitou and Nei 1987) with MEGA4 (Tamura et al. 2007).

### Chromosomal location and expansion patterns of Populus HAK genes

To categorize expansion of the *KT/HAK/KUP* gene family, we examined the chromosomal locations of all members of this family in Poplar (JGI v2.0). Segmental (chromosomal segments) duplication, tandem duplication (duplications in a tandem pattern), and transposition events result in gene family expansion (Cannon et al. 2004). In this study, we focused on the segmental and tandem duplication. Previous analysis of the Populus genome has identified paralogous segments created by the whole-genome duplication event in the Salicaceae (salicoid duplication), 60 to 65 million years ago (Tuskan et al. 2006). A method similar to that of Maher et al. (2006) was used to identify segmental duplications. First, the distributions of *KT/HAK/KUP* genes relative to Tuskan's duplicate genomic blocks (Tuskan et al. 2006) are illustrated using SyMAP (Synteny Mapping and Analysis Program) v3.3 (Soderlund et al. 2006). Next, 10 protein-coding genes upstream and downstream of each pair of paralogs were obtained from the annotated genomes of Poplar (JGI v2.0). Finally, the similarity between the genes flanking one *KT/HAK/KUP* gene and those flanking the other *KT/HAK/KUP* gene in each pair of paralogs was determined. Thus, we recognized that paralogous *KT/HAK/KUP* genes are originated from a duplication event if they resided within a region of conserved protein-coding genes.

### Exon structure and protein domain of HAK genes

Gene intron/exon structure information of alga, arabidopsis, rice, and Poplar HAK genes was collected from the genome annotations of Phytozome v6.0 (http://www.phytozome.net/), and a web application named Gene Structure Display Server (GSDS) (http://gsds.cbi.pku.edu.cn/) was used to draw gene structure diagram. Exon boundaries within the coding regions of each tandem or segmental duplicated Populus *KT/HAK/KUP* pairs were determined according to the 31st release of the Gramene database (http://www.gramene.org/). The numbers of nucleotides (nt) for each exon as well as the phase of each splicing site also were determined. The potential domains of each HAK protein was identified based on the Pfam database (Finn et al. 2008) to better understand its evolution within different species.

### Ratio of nonsynonymous to synonymous substitutions (dN/dS) and relative evolutionary rate test

The coding sequences of the Poplar HAK genes were aligned following the amino acid alignment by CodonAlign 2.0 (http://homepage.mac.com/barryghall/CodonAlign.html). The numbers of nonsynonymous nucleotide substitutions per nonsynonymous site (dN) and the numbers of synonymous nucleotide substitutions per synonymous site (dS) were estimated with the yn00 program of PAML4 (Yang 2007). Tajima relative rate tests (Tajima 1993) were performed with amino acid sequences for the 11 Poplar *KT/HAK/KUP* duplicate pairs using MEGA4 (Tamura et al. 2007).

## Results

### Identification and phylogenetic analysis of Populus *KT*/*HAK*/*KUP* genes

A total of 31 full-length genes encoding putative KT/HAK/KUP proteins were identified in the *P. trichocarpa* genome (Table 1). The number was larger than that of KT/HAK/KUP genes present in the rice (27 HAK genes) and almost 2.5 times to that of Arabidopsis (13 HAK genes) genomes. In addition to 21 *KT/HAK/KUP* genes identified by Yang et al. (2009), 10 genes also encoded *KT/HAK/KUP* potassium transporter, which was named as *PtHAK22* to *PtHAK31*, respectively. Two genes (*PtHAK23* and *PtHAK30*), each with 2 JGI loci, were re-predicted using FGENESH+ 2.6 software. The length of KT/HAK/KUP transporters in Poplar ranged from 258 aa to 860 aa, and the number of exons ranged from 1 to 11. The results of EST searches showed that all the *KT/HAK/KUP* genes in Poplar matched at least one significant EST hit (score > 400, *E < 1e-10*). These results indicate that all the *KT/HAK/KUPs* were expressed in Poplar genome.

**Table 1:** KT/HAK/KUP genes identified in the *Populus trichocarpa* genome

Gene name	Gene locus	Genome location	No. of exons	Transcript length (bp)	Peptide length (aa)	EST hit
PtHAK19	POPTR_0001s00580	Chromosome 1: 471,744- 477,819	9	2815	791	7
PtHAK1	POPTR_0001s00590	Chromosome 1: 484,931-491,430	8	3332	798	8
PtHAK4	POPTR_0001s03680	Chromosome 1: 2,943,939-2,948,721	10	2554	792	6
PtHAK22	POPTR_0001s12780	Chromosome 1: 9,892,455-9,896,030	8	1988	612	7
PtHAK20	POPTR_0001s12790	Chromosome 1: 9,913,205-9,918,209	10	2361	786	6
PtHAK23	POPTR_0001s15000/POPTR_0001s15010	Chromosome 1: 11,828,207-11,834,915	10	2442	814	5
PtHAK10	POPTR_0001s21310	Chromosome 1: 19,695,606-19,703,449	10	3023	860	4
PtHAK2	POPTR_0002s23850	Chromosome 2: 21,321,256-21,326,804	9	2799	785	4
PtHAK5	POPTR_0003s01820	Chromosome 3: 1,693,608-1,698,071	5	2051	455	4
PtHAK6	POPTR_0003s10910	Chromosome 3: 11,766,950-11,772,428	8	2403	764	7
PtHAK11	POPTR_0003s10920	Chromosome 3: 11,775,688-11,779,749	9	2541	846	7
PtHAK12	POPTR_0003s13370	Chromosome 3: 13,766,155-13,771,915	9	3090	776	4
PtHAK17	POPTR_0003s14800	Chromosome 3: 14,772,374-14,777,395	9	2329	731	4
PtHAK24	POPTR_0005s09870	Chromosome 5: 6,957,199-6,960,081	9	2133	645	4
PtHAK25	POPTR_0007s08130	Chromosome 7: 6,598,645-6,599,576	2	777	258	1
PtHAK21	POPTR_0008s14040	Chromosome 8: 9,273,295-9,278,893	8	2624	752	2
PtHAK13	POPTR_0008s14660	Chromosome 8: 9,733,881-9,740,180	11	2558	767	6
PtHAK14	POPTR_0008s14670	Chromosome 8: 9,742,547-9,749,434	8	2795	821	7
PtHAK26	POPTR_0009s07760	Chromosome 9: 7,210,167-7,215,402	8	2910	557	7
PtHAK3	POPTR_0010s10440	Chromosome 10: 10,946,928-10,952,709	8	2859	780	11
PtHAK18	POPTR_0010s10450	Chromosome 10: 10,957,148-10,963,581	9	2936	847	6
PtHAK27	POPTR_0010s11100	Chromosome 10: 11,462,692-11,467,835	8	2247	748	0
PtHAK28	POPTR_0012s04050	Chromosome 12: 3,265,446-3,266,562	1	1117	369	2
PtHAK29	POPTR_0012s04060	Chromosome 12: 3,277,356-3,282,179	9	1338	445	2
PtHAK8	POPTR_0013s08110	Chromosome 13: 7,052,758-7,057,838	10	2733	792	3
PtHAK15	POPTR_0014s12700	Chromosome 14: 9,342,456-9,347,605	8	2599	774	2
PtHAK7	POPTR_0014s14180	Chromosome 14: 10,376,641-10,382,102	9	2767	784	4
PtHAK9	POPTR_0015s05040	Chromosome 15: 5,287,410-5,292,962	8	2274	757	3
PtHAK16	POPTR_0019s08430	Chromosome 19: 9,958,198-9,963,729	10	2778	793	3
PtHAK30	POPTR_0327s00200/POPTR_0327s00210	Scaffold_327: 1,965-7,944	6	1920	639	4
PtHAK31	POPTR_0583s00210	Scaffold_583: 10,696-12,947	4	1547	511	3

*PtHAK23* and *PtHAK30* were re-predicted using FGENESH+ 2.6 software with the similarity. The EST searches for *HAK* genes in *P. trichocarpa* were performed using BLASTN tool (score > 400 e < 1e-10) against the EST database in PopulusDB.

**Figure 1:**
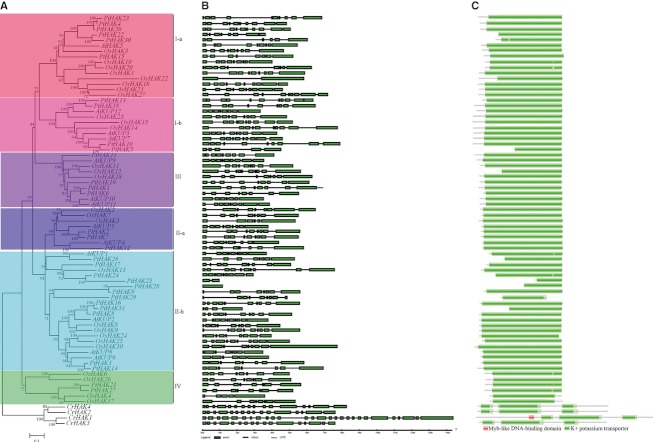
Phylogenetic analysis (A), exon/intron (B), and protein domain structure (C) of the KT/HAK/KUP gene family in *Populus trichocarpa*, *Arabidopsis thaliana*, green alga (*Chlamydomonas reinhardtii*), and rice (*Oryza sativa L*.). The bootstrap consensus phylogenetic tree was constructed with the green alga KT/HAK/KUP (CrHAK1-CrHAK4) protein as outgroup using the neighbor-joining method in MEGA4, and the numbers indicate the percentage bootstrap support. The evolutionary distances were computed using the Poisson correction method and are in the units of the number of amino acid substitutions per site. The green boxes and gray lines in the gene structure diagram (drawn to scale as indicated on bottom) represent exons and introns, respectively. Protein domains are shown as boxes based on identification by Pfam.

To gain more insight into evolution of the *KT/HAK/KUP* gene family in *P. trichocarpa*, the previously genome-wide identified amino acid sequences of 13 *KT/HAK/KUP* genes in dicotyledonous *Arabidopsis thaliana*, and 27 in monocotyledonous rice (*Oryza sativa* L.) (Table S1) were used in our phylogenetic analysis. Also, four genes in green alga (*Chlamydomonas reinhardtii*) were used as outgroup in our study. Phylogenetic analysis defined clearly the six KT/HAK/KUP groups (Fig. 1), namely I-a, I-b, II-a, II-b, III, and IV group. The classification in this analysis was consistent with the result of Banuelos et al. (2002) and Yang et al. (2009). The *KT/HAK/KUP* genes from each of the groups were found in all three studied species of the higher plants with the exception of members of group IV, which was absent in the Arabidopsis genome. The II-b group constituted the largest clade containing 12 Poplar KT/HAK/KUP members, and the I-a group formed the second largest clade containing six Poplar members. Additionally, the I-a and I-b groups further clustered forming a larger clade and implying that they originated from a common ancestor by subsequent gene duplication. Also, the same as II-a and II-b groups. Structural analyses can provide valuable information concerning duplication events when interpreting phylogenetic relationships within gene families. Thus, the exon/intron structure of each member of the KT/HAK/KUP family was analyzed (Fig. 1B). Most of the *KT/HAK/KUP* genes in high plant comprise 8–10 exons (less than that in green alga) and a last exon with the maximum length. Most members within the individual groups shared similar intron/exon structures, consistent with the phylogenetic classification. For all KT/HAK/KUP protein family members, K^+^ potassium transporter domain is present in all species with a high degree of similarity (Fig. 1C) except CrHAK1's Myb-like DNA-binding domain, indicating the functional conservation features of these protein. But some truncated K^+^ potassium transporter domain are found in some KT/HAK/KUP proteins, indicating a functional divergence to some extent of this protein family members during the long evolution process.

### Genomic organization of the Populus *KT*/*HAK*/*KUP* gene family

**Figure 2:**
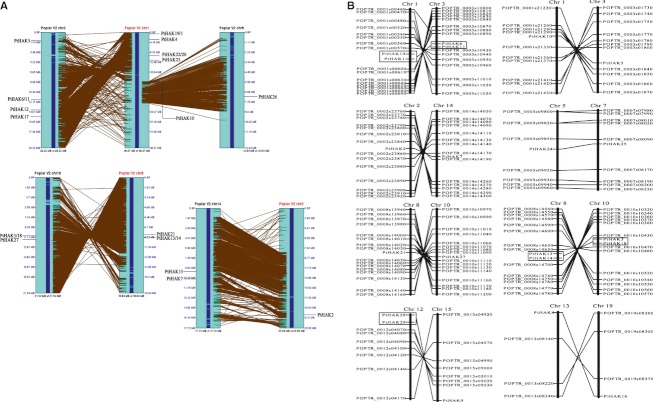
Chromosomal location (A) and paralogon analysis (B) of Populus KT/HAK/KUP genes. The distributions of HAK genes relative to Tuskan's duplicate genomic blocks (Tuskan et al. 2006) are illustrated using SyMAP (Synteny Mapping and Analysis Program) v3.3 (Soderlund et al. 2006). Ten protein-coding genes upstream and downstream of each pair of paralogs were obtained from the annotated genomes of Poplar (JGI v2.0), and the similarity between the genes flanking one HAK gene and those flanking the other HAK gene in each pair of paralogs was determined.

The physical locations of 29 *KT/HAK/KUP*s were dispersed to 13 of the 19 Populus chromosomes, while the other two HAK genes were localized to unassembled genomic sequence scaffolds and thus were not mapped to any particular chromosome (Table 1). The distribution of the *KT/HAK/KUP* genes among the chromosomes appears to be uneven: chromosomes 4, 6, 11, 16, 17, and 18 harbor no *KT/HAK/KUP* genes, while relatively high densities of *KT/HAK/KUP*s with some apparent tandem duplications were discovered in some locations on chromosomes 1 (seven genes) and 3 (five genes), and each of chromosomes 2, 5, 7, 9, 13, and 19 possess a single HAK gene (Table 1). Two paralogs located in tandem in a chromosomal segment are likely the result of single-gene duplication (Olinski et al. 2006). According to this, six pairs of tandem duplicated paralogs (PtHAK1/PtHAK19, PtHAK20/PtHAK22, PtHAK6/PtHAK11, PtHAK13/PtHAK14, PtHAK3/PtHAK18, PtHAK28/PtHAK29) were discovered in 31 Poplar HAK genes (Fig. 2).

The identification of chromosomal homologous segments within genomes (known as paralogons) can aid in studying genome evolution, such as genome duplication and rearrangement (Murphy et al. 2005). Paralogous segments of Populus genome created by the whole-genome duplication event in the Salicaceae (salicoid duplication), 60 to 65 million years ago, have been identified (Tuskan et al. 2006). The distributions of *KT/HAK/KUP* genes relative to the duplicate genomic blocks are illustrated in Figure 2A. Of the 31 genes, only nine are located outside any duplicate blocks. Next, 10 protein-coding genes upstream and downstream of each pair of paralogs were obtained from the annotated genomes of Poplar. Finally, the similarity between the genes flanking one HAK gene and those flanking the other HAK gene in each pair of paralogs was determined. Eight pairs of segmental duplicated paralogs (PtHAK1[19]/PtHAK6[11], PtHAK10/PtHAK5, PtHAK2/PtHAK7, PtHAK24/PtHAK25, PtHAK21/PtHAK27, PtHAK13[14]/PtHAK18[3], PtHAK28[29]/PtHAK9, PtHAK8/PtHAK16) were discovered in 31 Poplar *KT/HAK/KUP* genes, suggesting the ancestral relationship of these chromosomes or chromosome fragments.

### Exon structure and splice site analysis

**Figure 3:**
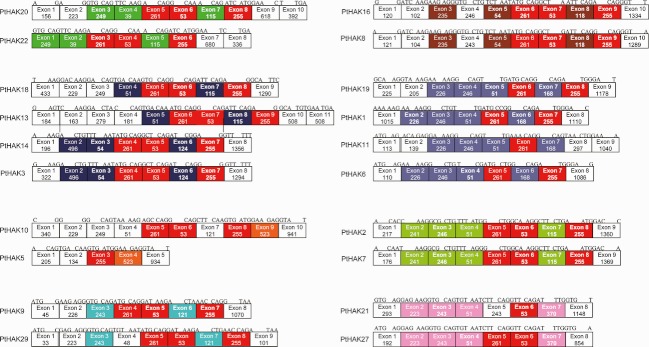
Exon structures (5'→3') and splice site analysis of each tandem or segmental duplicated Populus KT/HAK/KUP pairs. The exons that transverse or flank the splice sites are indicated above each exon boundary. Numbers in boxes are nucleotide length. Exons colored red are conserved in length among all Populus KT/HAK/KUP genes. Exons colored green, brown, darkblue, blue, gray, orange, lime, aqua, or rose are conserved in length between each duplicated Populus KT/HAK/KUP pairs. The size of each exon is not drawn to scale.

Conserved exon structures including exons with the same numbers of nucleotides as well as the conserved intron phases indicate similarities of the studied genes (von Schantz et al. 2006). Exons colored red are conserved in length and almost the same splice phase among all Populus HAK genes, including 261-nt exon, 53-nt exon, and 255-nt exon (Fig. 3). In addition to these three exons, there are equal-length-exons with the same splice phase shared by each duplicated HAK pairs. At least five conserved exon structure was shared by the tandem or segmental duplicated HAK pairs except two shared by PtHAK5/PtHAK10 pairs (Fig. 3), supporting the common ancestral relationship of these duplicated pairs.

### Evolution of the coding sequences of the 11 duplicated *KT*/*HAK*/*KUP* pairs

Modes of selection can be estimated by the ratio of the numbers of nonsynonymous substitutions per nonsynonymous site (dN) to the numbers of synonymous substitutions per synonymous site (dS), that is, dN/dS > 1 indicates positive selection; dN/dS < 1, purifying selection; and dN/dS = 1, neural selection (Yang and Nielsen 2000). The combination of phylogenetic, exon structure and splice site, and paragon analyses revealed 11 pairs of Poplar *KT/HAK/KUP* duplicates (Table 2). The nonsynonymous substitution rates of all these 11 duplicate pairs are markedly lower than their synonymous substitution rates and their dN/dS values are <1 (Table 2), suggesting that these duplicates likely have been subject to purifying selection. Furthermore, Tajima (Tajima 1993) relative rate tests were conducted to investigate whether one of the *KT/HAK/KUP* duplicates has evolved at an accelerated rate following the duplication. A statistically significant increase in evolutionary rate occurs in three segmental duplicated pairs (PtHAK1/PtHAK6, PtHAK13/PtHAK18, PtHAK24/PtHAK25) (Table 3). These eight pairs are included in the Group I-b, II-b, and III in our phylogenetic analysis.

**Table 2:** Divergence between paralogous KT/HAK/KUP gene pairs in Populus

No	Gene 1	Gene 2	dN	dS	dN/dS
1O	PtHAK23	PtHAK4	0.0049 ± 0.0035	0.0219 ± 0.0127	0.2248
2O	PtHAK22	PtHAK30	0.1614 ± 0.0119	0.1767 ± 0.0216	0.9132
3S	PtHAK13	PtHAK18	0.0907 ± 0.0156	0.2917 ± 0.0578	0.3108
4S	PtHAK10	PtHAK5	0.0445 ± 0.0106	0.2409 ± 0.0513	0.1849
5S	PtHAK1	PtHAK6	0.0123 ± 0.0055	0.1965 ± 0.0441	0.0627
6S	PtHAK2	PtHAK7	0.0150 ± 0.0062	0.3348 ± 0.0628	0.0449
7S	PtHAK24	PtHAK25	0.1157 ± 0.0181	0.2378 ± 0.0473	0.4864
8S	PtHAK28	PtHAK9	0.0476 ± 0.0110	0.2786 ± 0.0571	0.1709
9O	PtHAK16	PtHAK31	0.0000 ± 0.0000	0.0067 ± 0.0067	0
10S	PtHAK3	PtHAK14	0.0257 ± 0.0082	0.2532 ± 0.0507	0.1015
11S	PtHAK21	PtHAK27	0.0583 ± 0.0123	0.1857 ± 0.0420	0.3141

These gene pairs were identified at the terminal nodes of the gene tree shown in Figure 1. Gene pairs created by tandem duplication (T), segment duplication (S), or other (O) events are indicated in the first column of the table. Synonymous (dS) and nonsynonymous substitution (dN) rates are presented for each pair.

**Table 3:** Tajima relative rate tests of Populus KT/HAK/KUP duplicate genes^a^

Testing group	Mt^b^	M1^c^	M2^d^	X^2^	*P*^e^
*PtHAK23*/*PtHAK4* with *AtHAK5*	458	7	3	1.60	0.20590
*PtHAK22*/*PtHAK30* with *AtHAK5*	295	10	9	0.05	0.81855
*PtHAK16*/*PtHAK31* with *AtKUP2*	392	1	0	1.00	0.31731
*PtHAK1*/*PtHAK6* with *AtKUP10*	606	10	28	8.53	0.00350
*PtHAK3*/*PtHAK14* with *AtKUP6*	610	7	14	2.33	0.12663
*PtHAK10*/*PtHAK5* with *AtKUP7*	355	17	17	0.00	1.00000
*PtHAK2*/*PtHAK7* with *AtKUP3*	613	20	17	0.24	0.62187
*PtHAK24*/*PtHAK25* with *OsHAK13*	161	7	27	11.76	0.00060
*PtHAK21*/*PtHAK27* with *OsHAK6*	353	8	12	0.80	0.37109
*PtHAK13*/*PtHAK18* with *AtKUP12*	572	50	14	20.25	0.00001
*PtHAK28*/*PtHAK9* with *OsHAK13*	152	11	7	0.89	0.34578

a The Tajima relative rate test was used to examine the equality of evolutionary rate between Populus duplicate pairs.

b Mt is the sum of the identical sites and the divergent sites in all three sequences tested.

c M1 is the number of unique differences in the first paralog.

d M2 is the number of unique differences in the second paralog.

e If *P* < 0.05, the test rejects the equal substitution rates between the two duplicates and infers that one of the two duplicates has an accelerated evolutionary rate.

## Discussion

Gene duplication including segmental (chromosomal segments) duplication, tandem duplication (duplications in a tandem pattern), and transposition events can result in gene family expansion (Cannon et al. 2004), and functional diversification among gene family members have been considered as an important source of evolutionary innovation in complex organisms. Lineage-specific expansion of *KT/HAK/KUP* gene families in rice has been reported (Yang et al. 2009). However, there is far less information about this family for woody plant species. Although 21 Poplar *KT/HAK/KUP* genes were mentioned in rice's comparative genomic study (Yang et al. 2009), only part of *KT/HAK/KUP* genes have been identified because of the authors focus and sequenced gap of *P. trichocarpa* genome database (JGI v1.0), and no evolutionary information, expansion pattern, and modes of selection about this family was mentioned. In our study, through genome-wide in-depth investigation, we identified 31 members of KT/HAK/KUP potassium transporter gene family in Poplar genome, larger members than that in rice, and more than twice of the members in *Arabidopsis*. To investigate the expansion of KT/HAK/KUP family in Poplar, a phylogenetic tree was reconstructed using plant full-length KT/HAK/KUP proteins. The phylogenetic tree divided the plant *KT/HAK/KUP* genes into six distinct groups, and Populus *KT/HAK/KUP* genes were dispersed to all groups. Paralogous segments of Populus genome created by the whole-genome duplication event in the Salicaceae (salicoid duplication), 60 to 65 million years ago, have been identified (Tuskan et al. 2006). Of the 31 genes, only nine are located outside of any duplicate genomic blocks. Eight pairs of paralogous genes were found, suggesting that segmental duplication of genes was the major force for expansion of the KT/HAK/KUP family in Poplar. It should be noted that six pairs of tandem duplicated paralogs (PtHAK1/PtHAK19, PtHAK20/PtHAK22, PtHAK6/PtHAK11, PtHAK13/PtHAK14, PtHAK3/PtHAK18, PtHAK28/PtHAK29) were discovered in 31 Poplar *KT/HAK/KUP* genes. Of these six pairs, four pairs (PtHAK13/PtHAK14, PtHAK3/PtHAK18, PtHAK1/PtHAK19, and PtHAK6/PtHAK11) were also found in segmental duplications. We speculate segmental duplications might occur before tandem duplications as far as these four pairs. Maybe four HAK genes were descended from two ancestral HAK genes through segmental duplication, and then tandem duplications occurred leading to four pairs (eight genes) present in the current Poplar genome. Further investigation suggests that segmental duplication and tandem duplication all contributed to the expansion of this family in Poplar. Such conclusion presented here is further supported by the high degree of conservation of exon structures (Fig. 2) and paralogon analysis (Fig. 3).

After gene duplication, two duplicates can undergo substantial functional divergence, and it plays a major role in the evolution of new functions (Ohta 1989). In this study, the combination of phylogenetic, exon structure and splice site, and paragon analyses revealed 11 pairs of Poplar KT/HAK/KUP duplicates, and purify selection has been found to contribute to the evolution of these pair. These results suggested that purify selection contributed to evolution of this gene family. Furthermore, Tajima relative rate tests identified accelerated evolutionary rates in three of the duplicates (Table 3), but none of the three pairs reported to have dN/dS > 1. Therefore, the rate increases might simply be at silent sites which do not contribute to functional diversification.

In summary, 31 Poplar *KT/HAK/KUP* transporter genes have been identified in genome-wide analysis, and segmental duplication and tandem duplication events contributed to the expansion of this family in Poplar. Purify selection and asymmetric evolutionary rates have been found to contribute to the evolution of this gene family. With such in-depth investigation, it might provide more insights into the underlying evolution mechanisms of trees acclimating to their natural habitat.

## Supporting Information

**Table S1.** KT/HAK/KUP potassium transporter gene family genes in *Arabidopsis thaliana, Oryza sativa L.*, and *Chlamydomonas reinhardtii*
